# Supplementary material for: Determinants of effective interventions for HIV prevention, treatment, and care to address inequitable HIV outcomes among Black Women of African Descent (BWAD) in High-Income Countries: Systematic review protocol
Source: PLoS One. 2024 Jun 11;19(6):e0304255. doi: 10.1371/journal.pone.0304255 (PMC11166283; doi:10.1371/journal.pone.0304255)
Supplement: S2 File — (DOCX) [file pone.0304255.s002.docx]

**Determinants of effective interventions for HIV prevention, treatment, and care to address inequitable HIV outcomes among Black Women of African Descent (BWAD) in High-Income Countries: Systematic review protocol.**

**Additional File 2. Search Terms**

Search Terms: Medline (OVID)

**Set 1: Intervention**

1. *MeSH*

HIV prevention and Pre-exposure prophylaxis adj2 (Initiative* or Service* or Program* or Intervention*)

HIV treatment and care adj2 (diagnosis or antiretroviral therapy or ART or Testing or Counseling) or 2(Initiative* or service* or Program* or Intervention*)

1. *Keyword*

HIV prevention (Education, awareness creation, Knowledge creation), Pre-exposure prophylaxis

*C) Keyword*

HIV treatment and care/ or exp Antiretroviral Therapy /or ART/ or exp HCT Testing or Counseling/ or exp Pre-exposure prophylaxis.

**Set 2: Population**

1. *MeSH*

High-Income Country*

1. *Keyword*

Andorra or Antigua and Barbuda or Aruba or Australia or Austria or The Bahamas or Bahrain or Barbados or Belgium or Bermuda or British Virgin Islands or Brunei Darussalam or Canada or Cayman Islands or Channel Islands or Chile or Croatia or Curacao or Cyprus or Czech Republic or Denmark or Estonia or Faroe Islands or Finland or France or French Polynesia or Germany or Gibraltar or Greece or Greenland or Guam or Hong Kong SAR, China or Hungary or Iceland or Ireland or Isle of Man or Israel or Italy or Japan or Korea, Rep. or Kuwait or Latvia or Liechtenstein or Lithuania or Luxembourg or Macao SAR or China or Malta or Mauritius or Monaco or Nauru or Netherlands or New Caledonia or New Zealand or Northern Mariana Islands or Norway or Oman or Palau or Panama or Poland or Portugal or Puerto Rico or Qatar or Romania or San Marino or Saudi Arabia or Seychelles or Singapore or Sint Maarten or Slovak Republic or Slovenia or Spain or St Kitts and Nevis or St Martin or Sweden or Switzerland or Trinidad and Tobago or Turks and Caicos Islands or United Arab Emirates or United Kingdom or United States or Uruguay or Virgin Islands )

**Set 3: Search Alone**

*A) MeSH*

Black Women or girls and Africa* or Caribbean

Black Women/

*B) Keyword*

Wom?n or or female*

**Set 4: Study Design**

Qualitative Research/ or Quantitative Research / or Mixed research/ or multi level research

**Set 5: Outcomes: N/A**

**Search Summary:** Set 1 (A or B or C) and Set 2 (A or B) and Set 3 (A or B) and Set 4
