# Supplementary material for: Determinants of effective interventions for HIV prevention, treatment, and care to address inequitable HIV outcomes among Black Women of African Descent (BWAD) in High-Income Countries: Systematic review protocol
Source: PLoS One. 2024 Jun 11;19(6):e0304255. doi: 10.1371/journal.pone.0304255 (PMC11166283; doi:10.1371/journal.pone.0304255)
Supplement: Attachment — Submitted filename: Response to Reviewers 24 Jan 2024.docx [file pone.0304255.s003.docx]

**PONE-D-23-33845: Rebuttal letter that responds to each point raised.**

Dear Editor, Dr. Yury E Khudyakov,

Thank you for giving us the opportunity to submit a revised draft of the manuscript PONE-D-23-33845: Determinants of effective interventions for HIV prevention, treatment, and care to address inequitable HIV outcomes among Black Women of African Descent (BWAD) in High-Income Countries: Systematic review protocol. We appreciate you and the reviewers for your precious time in reviewing our paper and providing valuable comments. The authors have carefully considered the comments and tried our best to address every one of them. We hope the manuscript after careful revisions meet your high standards. The authors welcome further constructive comments if any.

Below we provide the point-by-point responses, rebuttal letter that responds to each point raised by your self and reviewer(s). We uploaded all the documents including **'Response to Reviewers**; **'Revised Manuscript with Track Changes'** and an unmarked version of **'Manuscript'.**

If you would like to make changes to your financial disclosure, please include your updated statement in your cover letter. Guidelines for resubmitting your figure files are available below the reviewer comments at the end of this letter.

Response: Thank you for pointing out this point. We have already indicated why we are not submitting funding approval for this manuscript on the submitted cover letter: “As this protocol is based on the big and multisite study project, we will not share the proof of external funding letter of approval for now. If you need more information, please contact the fourth Author and the project Nominated Principal Applicant (NPA). Dr. J. Etowa, at the University of Ottawa: [jetowa@uottawa.ca](mailto:jetowa@uottawa.ca). Thank you for your understanding and consideration on this aspect.”

If applicable, we recommend that you deposit your laboratory protocols in protocols.io to enhance the reproducibility of your results. Protocols.io assigns your protocol its own identifier (DOI) so that it can be cited independently in the future. For instructions see: <https://journals.plos.org/plosone/s/submission-guidelines#loc-laboratory-protocols>. Additionally, PLOS ONE offers an option for publishing peer-reviewed Lab Protocol articles, which describe protocols hosted on protocols.io. Read more information on sharing protocols at <https://plos.org/protocols?utm_medium=editorial-email&utm_source=authorletters&utm_campaign=protocols>.

Response: Not applicable

Journal Requirements:

When submitting your revision, we need you to address these additional requirements.

1. Please ensure that your manuscript meets PLOS ONE's style requirements, including those for file naming. The PLOS ONE style templates can be found at 
<https://journals.plos.org/plosone/s/file?id=wjVg/PLOSOne_formatting_sample_main_body.pdf> and 
<https://journals.plos.org/plosone/s/file?id=ba62/PLOSOne_formatting_sample_title_authors_affiliations.pdf>

Response: Thank you for your advice. Our manuscript followed the guidelines.

2. Thank you for stating the following financial disclosure: 
"The research program is supported by the Canadian Institutes of Health Research (CIHR), Grant # FRN 183803. "
 
Please state what role the funders took in the study.  If the funders had no role, please state: ""The funders had no role in study design, data collection and analysis, decision to publish, or preparation of the manuscript.""

Response: Thank you for the suggestion, we have revised it accordingly, see page#12

If this statement is not correct you must amend it as needed.

Please include this amended Role of Funder statement in your cover letter; we will change the online submission form on your behalf.

Response: Thank you for the suggestion, the role of the funder was indicated on the cover letter.

3. Thank you for stating the following in the Acknowledgments Section of your manuscript: 
"The authors are grateful to Collaborative Critical Research for Equity and Transformation in
Health (CO-CREATH) Lab, School of Nursing, Faculty of Health Sciences, University of Ottawa for creating enabling situation for Black people health inequity critical research; and the Canadian 
Institutes of Health Research (CIHR) for providing funding for this study. "

We note that you have provided funding information that is not currently declared in your Funding Statement. However, funding information should not appear in the Acknowledgments section or other areas of your manuscript. We will only publish funding information present in the Funding Statement section of the online submission form. 
Please remove any funding-related text from the manuscript and let us know how you would like to update your Funding Statement. Currently, your Funding Statement reads as follows: 
"The research program is supported by the Canadian Institutes of Health Research (CIHR), Grant # FRN 183803. "

Please include your amended statements within your cover letter; we will change the online submission form on your behalf.

Response: Thank you for the suggestion, the funding information is declared under funding statement, please see on page #12, and the role of the funder was indicated on the cover letter.

4. Please note that in order to use the direct billing option the corresponding author must be affiliated with the chosen institute. Please either amend your manuscript to change the affiliation or corresponding author, or email us at plosone@plos.org with a request to remove this option.

Response: Thank you for your advice. Please use School of Nursing, Faculty of Health Sciences, University of Ottawa, Ottawa, Ontario, Canada for direct billing: please use the name of: Dr. J. Etowa, at the University of Ottawa: [jetowa@uottawa.ca](mailto:jetowa@uottawa.ca).

5. We note that you have stated that you will provide repository information for your data at acceptance. Should your manuscript be accepted for publication, we will hold it until you provide the relevant accession numbers or DOIs necessary to access your data. If you wish to make changes to your Data Availability statement, please describe these changes in your cover letter and we will update your Data Availability statement to reflect the information you provide.

Response: Thank you for you for pointing out this. Now we have included Data availability statement, please on page# 12: “Data availability statement: All data relevant to the study are included in the article or uploaded as supplementary information. Not applicable.”
**Additional Editor Comments:**

Your manuscript was reviewed by one expert in the field who identified many important problems in your submission and produced copious comments. It is essential that you provide thorough responses to all comments submitted by the reviewer.

[Note: HTML markup is below. Please do not edit.]

Reviewers' comments:

Reviewer's Responses to Questions

**Comments to the Author**

1. Does the manuscript provide a valid rationale for the proposed study, with clearly identified and justified research questions?

The research question outlined is expected to address a valid academic problem or topic and contribute to the base of knowledge in the field.

Reviewer #1: Yes

Thank you.

2. Is the protocol technically sound and planned in a manner that will lead to a meaningful outcome and allow testing the stated hypotheses?

The manuscript should describe the methods in sufficient detail to prevent undisclosed flexibility in the experimental procedure or analysis pipeline, including sufficient outcome-neutral conditions (e.g. necessary controls, absence of floor or ceiling effects) to test the proposed hypotheses and a statistical power analysis where applicable. As there may be aspects of the methodology and analysis which can only be refined once the work is undertaken, authors should outline potential assumptions and explicitly describe what aspects of the proposed analyses, if any, are exploratory.

Reviewer #1: Yes

Thank you.

3. Is the methodology feasible and described in sufficient detail to allow the work to be replicable?

Descriptions of methods and materials in the protocol should be reported in sufficient detail for another researcher to reproduce all experiments and analyses. The protocol should describe the appropriate controls, sample size calculations, and replication needed to ensure that the data are robust and reproducible.

Reviewer #1: Yes

Thank you.

4. Have the authors described where all data underlying the findings will be made available when the study is complete?

The [PLOS Data policy](https://journals.plos.org/plosone/s/materials-and-software-sharing) requires authors to make all data underlying the findings described in their manuscript fully available without restriction, with rare exception, at the time of publication. The data should be provided as part of the manuscript or its supporting information, or deposited to a public repository. For example, in addition to summary statistics, the data points behind means, medians and variance measures should be available. If there are restrictions on publicly sharing data—e.g. participant privacy or use of data from a third party—those must be specified.

Reviewer #1: Yes

Thank you.

5. Is the manuscript presented in an intelligible fashion and written in standard English?

PLOS ONE does not copyedit accepted manuscripts, so the language in submitted articles must be clear, correct, and unambiguous. Any typographical or grammatical errors should be corrected at revision, so please note any specific errors here.

Reviewer #1: No

Response: Thank you for your suggestion. The manuscript is fully revised by academic English professional.

6. Review Comments to the Author

Please use the space provided to explain your answers to the questions above and, if applicable, provide comments about issues authors must address before this protocol can be accepted for publication. You may also include additional comments for the author, including concerns about research or publication ethics.

You may also provide optional suggestions and comments to authors that they might find helpful in planning their study.

(Please upload your review as an attachment if it exceeds 20,000 characters)

Reviewer #1: Dear Authors, 
 
Thank you very much for your hard work and important study protocol for an extremely important subject. Thank you also for the opportunity to read and review your protocol. I believe your study has implications to better inform effectiveness of interventions for HIV prevention, treatment and care to BWAD to address inequitable HIV outcomes in HICs.

Overall, the protocol is good; however, there are several suggestions for sentence structure and grammar for your consideration and I do recommend another peer edit. Please ensure that you stick to one voice and not switch from active to passive voice (i.e., the authors… and then switch to “we will also…” – example in the Data management and extraction section). I have several comments, recommendations and suggestions for your consideration.  Once addressed, I believe the protocol and systematic review will a great contribution.
 
Response: Thank you for your suggestion. The manuscript is fully revised by academic English professional.
 
**Abstract:**
- Suggest rewording first sentence (for conciseness) to read: In High-Income Countries (HICs) HIV/AIDS continues to disproportionally affect Black Women of African Descent (BWAD) and other racialized groups.

Response: Thank you for suggestion. We have revised accordingly.
- Suggest rewording last sentence (for conciseness): This protocol outlines the methodological process of a systematic review that will gather quantitative and qualitative data to examine existing determinants of effective HIV prevention, treatment, and care interventions to address the HIV outcomes disparities and inequities among BWAD in HICs.
Response: Thank you for suggestion. We have revised accordingly.

**Background:** 
 
Paragraph 1:

- "Globally in the last four decades HIV, the virus that causes acquired immunodeficiency syndrome (AIDS) is continued to be one of the major public health concerns and health agenda.” Sentence is awkward. Which health agenda? Please clarify the sentence…is it SDGs? Hight income countries? Suggest: Globally in the last four decades HIV, the virus that causes acquired immunodeficiency syndrome (AIDS) continues to be a major public health concern….”

Response: Thank you for suggestion. We have revised accordingly.
- Racialized people including Black Women of African Descent (BWAD)(referred to as Black women and girls) continue to bear a disproportionate portion of the burden of HIV/AIDS and related l HIV-related outcomes (1,2,4). Sentence is awkward. Suggest "Racialized people, particularly Black Women of African Descent (referred to as Black women and girls) still disproportionately carry the burden of HIV/AIDS and its related outcomes."

Response: Thank you for suggestion. We have revised accordingly.

Paragraph 2:
- Globally, significant achievements have been registered in HIV prevention, treatment, support, and the reduction of its impact, however, in HICs racialized people including BWAD are still experiencing substantial HIV-related outcomes disparities. Sentence awkward. Suggest: Globally, significant achievements have been made in HIV prevention, treatment, support, and the reduction of its impact, however, in HICs racialized people, including BWAD, are still experiencing substantial HIV-related outcomes disparities.
Paragraph 3

Response: Thank you for suggestion. We have revised accordingly see on page#3.
- In the United Kingdom (UK), Ireland, and US, where declines in new HIV diagnoses have been smaller among Black people than among white populations. Suggest taking out “where” to read, “In the United Kingdom (UK), Ireland, and US, declines in new HIV diagnoses have been smaller among Black people than among white populations.”
- In this section, in general, suggest to starting off with general racialized populations, and then focusing on BWAD. Right now it jumps from one to another throughout. Maybe establish the disparities among racialized and Indigenous communities and then focus on BWAD? What do you think? As the population you are focusing on in your systematic review is BWAD.

Response: Thank you for suggestion. We have revised accordingly, see on page#3.

Paragraph 4 –
- Please provide a reference for your definition of health disparities.
- Review paragraph as there are some grammatical issues (periods in middle of second sentence (after age).
- The last sentence is quite long. Suggest dividing into two. Stop at “health outcomes (17).” Perhaps the next sentence should read, “They can be addressed by equity driven policy and practice…”. What is meant by policy and practice? Is it decisions, strategies? Please clarify.

Response: Thank you for suggestion. We have revised accordingly, see on page#4.

Paragraph 5
- Second sentence awkward, suggest “This disproportionately in high vulnerability to HIV is associated with intersecting social determinants, structural and systemic factors (i.e., racism, stigma) and inequitable access to health care that limits achievement of optimal health outcomes.”

Response: Thank you for suggestion. We have revised accordingly, see on page#4.
- Should be “BWAD experience multiple and intersecting… compared to non-racialized women.” in the 4th sentence. –

Response: Thank you for suggestion. We have revised accordingly, see on page#4

Paragraph 6
- The following sentence is awkward. “Since the epidemic peaked, although the new HIV infections and AIDS-related deaths have markedly decreased, yet efforts have fallen short for many HICs to address the systematic differences in HIV outcomes among marginalized and racialized people like BWAD.” Not sure what you are meaning. Please clarify, is it that related deaths have decreased since the epidemic peaked (are you referring to the AIDS epidemic?) and yet BWAD continue to bear the burden of HIV/AIDS in HICs? If so, suggest:
“Although the new HIV infections and AIDS-related deaths have markedly decreased since the peak of the AIDS pandemic, efforts by HICs to address the systematic differences in HIV outcomes among marginalized and racialized people like BWAD have fallen short.”?
- Suggest rewriting this sentence: “The existing interventions are critical in preventing new HIV infection and helping people living with virus, however the existing HIV programs are insufficient to reach the UNAIDS 95-95-95 targets and ending the health threat of HIV/AIDS in HICs(1,2) by 2030….. among black and other racialized women” Is this what you are getting at? “The existing interventions are critical in preventing new HIV infection and helping people living with virus, however they are insufficient to reach the UNAIDS 95-95-95 targets and ending the health threat of HIV/AIDS in HICs (1,2) by 2030. The racial and ethnic implications leading to inequities faced by BWAD and racialized women suggest system wide fragmentation regarding HIV prevention programing and policies.”

Response: Thank you for suggestion. We have revised accordingly, see on page#6

Paragraph 7
- Awkward wording in the second sentence (‘a growing number of evidence have underscored the need…”). Suggest something like…” "Over the past four decades, unchanged HIV intervention approaches have highlighted an increasingly urgent need for a tailored, innovative, and equitable approach to effectively end the HIV epidemic to address inequalities in HIV-related health outcomes."

Response: Thank you for suggestion. We have revised accordingly, see on page#6

Overall comment about the introduction:
You have done a great job providing evidence and rationale behind the disparities faced by racialized women, particularly among BWAD in HICs. The way the introduction is written, you are jumping back and forth between racialized women and then BWAD and sometimes looking at both. Since your research questions is focused on BWAD in particular, may I suggest a funnel system that starts off broadly about HIV, then talk about inequities faced by racialized and Indigenous communities globally, then racialized and Indigenous women globally, then focus on HICs in particular, then focus on BWAD only as that is your focus in this systematic review. What do you think?

Another thought in reading the introduction is the provision of the definitions of health disparities and health inequities in paragraph 4 which almost come out of nowhere and cut the introduction. Suggest somehow, weaving this into the beginning in terms of globally what health dipartites and inequities are and then move into how these impact racialized and Indigenous communities. Whichever way you think flow would be better, as now it seems to cut the flow of thought while reading it.

Response: Thank you for suggestion. We have revised accordingly line by line.

Systematic Review Question

Currently reads: What are the determinants of effective interventions for HIV prevention, treatment, and care [intervention/comparator] to improve HIV and related health outcomes [outcome] among Black Women of African Descent (BWAD) in HICs [population]? The question is clear in terms of your intervention, population and outcome. The sub-questions provided are well outlined for qual, quant and mixed methods studies. Please note question #3 seems to have “programsthat” instead of “programs that”.

Response: Thank you for your suggestion. We have revised accordingly, see on page #6

Suggest the following layout for conciseness as there seems to be some overlap in information provided:
- Under study design you describe BWAD and types of studies included. Suggest leaving the population description to the population section in the inclusion criteria aas quite repetitive.
- I would title the first section Protocol registration and reporting and put the section about PROSPERO and PRISMA here right after the major Methods section.
- Next is Inclusion criteria, this is where you describe the population, intervention/exposure, outcomes of interest and types of studies to be included [which is how you have it set up].
- Under Outcomes: In your RQ, you use the word “effective interventions” which insinuates some measurable outcome. How will this be defined? Are there specific primary or secondary interventions outcomes you will be looking at in addition to the sub-questions that focus on the types of interventions, barriers and facilitators and lessons learned. If you use effective, I think you will have to define what you mean by this? What have other protocols defined effective as? Are there examples of outcome variables that showed better health outcomes for BWAD or racialized communities? If so, what are those? Suggest looking at existing protocols/studies that outline effective strategies to guide you.

Response: Thank you for pointing out this and your suggestion. We have revised accordingly, see on page #8

Table 1: your population states “Adults (16 years or older) who have experienced DSVA; adults who have
perpetrated DSVA”. The question only had adults who experienced DSVA? Should you change it to experience and perpetrate DSVA? It is noted later that you outline (line 170) that experience of DSVA “either as someone who has experienced or perpetrated DSVA”. Since this is not clear from the onset, when reading the question, the phrasing insinuates services/interventions supporting individuals who have experienced DSVA which does not necessarily intuitively include perpetrators. Clarity is needed for the systematic review process and in the question. In the section on intervention/exposure, you list classes of interventions, what were the outcomes of those that you could look at to determine effective ones and how are they deemed effective. I hesitate to leave this section without any examples of possible outcome measures to include in a systematic review. Additional information would provide the reader with a better idea as to what these outcomes are. In this systematic review, will there be measured effects in the quantitative studies? Will these be outlined?

Response: Sorry we did not understand how this comment applies to our protocol, as we didn’t include any information related to “Adults (16 years or older) who have experienced DSVA”

Inclusion/exclusion of studies
- What about letters/commentaries and conference abstracts?
- Indicated only non-experimental; will there be experimental, quasi-experimental, randomized, quasi-randomized trials/studies? You mentioned quantitative, so only non-experimental quantitative. Please clarify.

Search Strategy:
- You have a decent # of databases so am sure you will catch many. Did you consider Gender Watch (ProQuest) [gender and women's studies; higher risk groups] or Web of Science?
- Will there be a grey literature search through google/google scholar? Please clarify rationale for not including grey literature or book chapters/programme reports that maybe relevant.

Response: Thank you for highlighting this: We organized the search strategies including the data base selection. They suggested the mentioned data bases MEDLINE(R) ALL (Ovid), Embase (Ovid), CINAHL (EBSCO Host), and Global Health (Ovid) are comprehensive to conduct the search for peer-reviewed studies.

Certainty of Cumulative Evidence:
- Please clarify, you will use the GRADE-CERQual for qualitative studies and GRADE approach for the quantitative studies?
- Additionally, the citations for both are not the original citations for the GRADE-CERQual and GRADE approach, they are citations of the authors’ own papers when they completed or used this approach. Please use the original citations –
o <https://www.cerqual.org/guide-for-decision-makers/>
o <https://training.cochrane.org/handbook/current/chapter-14>

Response: Thank you for pointing out this and your suggestion. We have addressed accordingly, please see page#10

Appraisal of study quality
- Which quality appraisal tool will be used? JBI? CASP? Cochrane ROBINS-I? Please provide rationale as to why this hasn’t been decided.

Response: Thank you for pointing out this and your suggestion. The methodological quality assessments of studies will be performed using Joanna Briggs Institute critical appraisal checklists. see on page #10

Discussion:
- Suggest adding a sentence in the discussion that any changes to the protocol will be updated on PROSPERO and final manuscript.

Response: Thank you for pointing out this and your suggestion. See page# 11

References:
- Note you have the title References twice.

Response: Thank you for pointing out this, now addressed.

7. PLOS authors have the option to publish the peer review history of their article ([what does this mean?](https://journals.plos.org/plosone/s/editorial-and-peer-review-process#loc-peer-review-history)). If published, this will include your full peer review and any attached files.

If you choose “no”, your identity will remain anonymous but your review may still be made public.

**Do you want your identity to be public for this peer review?** For information about this choice, including consent withdrawal, please see our [Privacy Policy](https://www.plos.org/privacy-policy).

Reviewer #1: No
