# Supplementary material for: Determinants of effective interventions for HIV prevention, treatment, and care to address inequitable HIV outcomes among Black Women of African Descent (BWAD) in High-Income Countries: Systematic review protocol
Source: PLoS One. 2024 Jun 11;19(6):e0304255. doi: 10.1371/journal.pone.0304255 (PMC11166283; doi:10.1371/journal.pone.0304255)
Supplement: Attachment — Submitted filename: Response to Reviewers 12 March 2024.docx [file pone.0304255.s004.docx]

Response to Reviewers: March 12, 2024

Dear Dr. Muhammad Shahzad Aslam,

Thank you for reviewing and your positive feedback on our manuscript.

Now, we have addressed all concerns of the reviewers, please see uploaded documents asper your advice.

Thank you for your continued understanding and Support,

Akalewold MSc, PhD©

|  | Reviewers comment | Response form authors |
| --- | --- | --- |
| Reviewer #1: | Reviewer #1: Well done on the review of the protocol. The authors have addressed all my comments. One small suggestion for the revised version - under the exclusion criteria, the sentence "Studies don not reporting the full finding article/manuscript report like conference abstracts" reads awkwardly, requires minor editing. Great and important work and I look forward to reading the final systematic review. | Thank you for your positive feedback. Thank you for bringing this to our attention, now we have revies this accordingly. Page#7 |
| Reviewer #2: | The only major concern is that this proposal is indicating a series of studies, while one same research may contribute information to different sub-analysis regarding different outcome. I.e., it is an umbrella proposal. Intervention strategy and outcome measure of each sub-analysis should be explicitly defined. I suggest authors may consider three points: | Thank you for your positive feedback. |
|  | 1) Describe scope of intervention strategies, and similarly, more specific outcome of interest. A cross-tab like combination table may help audience find specific topic of interest; | Thank you for your critical comment and bringing this to our attention. We already have described the scope of intervention using UNAIDS categorized HIV intervention in the inclusion criteria: Intervention/ Exposure context: “Eligible studies will involve standard specific interventions or combination HIV interventions in both community and in clinical settings. UNAIDS categorized HIV as a mix of different classes of interventions (behavioral, biomedical, and structural)(33). Behavioral HIV Prevention interventions include education, awareness creation, knowledge creation, pre-exposure prophylaxis (PrEP)/Post-exposure prophylaxis (PEP), biomedical HIV intervention includes diagnosis or HIV testing and counseling (HTC), HIV treatment and care such as Antiretroviral treatment (ART) and prevention of mother-to-child transmission of HIV (PMTCT); Structural HIV intervention include social and economic support aligned with HIV prevention, treatments, and care> Please see on page #7. |
|  | 2) Propose an example of a specific research target. E.g., effectiveness of PrEP on HIV incidence rate/diagnosis rate among BWAD; | Thank you for your suggestion. “We will analyze the included studies categories and outcome using UNAIDS category of HIV interventions (behavioral, biomedical, and structural)”, see page #7 and #10. |
|  | 3) Two distinct subgroups among BWAD may experience totally different social economic challenge and support. It will be the best to differentiate new immigrant BWAD from local-born BWAD community. | Thank you for this critical suggestion and for bringing this to our attention. Now we have addressed as follow : We will report the demographic characteristics of included study population including disaggregated “Foreign-born (Immigrant) and Non migrant of their country of residence. |
