# Supplementary material for: Determinants of effective interventions for HIV prevention, treatment, and care to address inequitable HIV outcomes among Black Women of African Descent (BWAD) in High-Income Countries: Systematic review protocol
Source: PLoS One. 2024 Jun 11;19(6):e0304255. doi: 10.1371/journal.pone.0304255 (PMC11166283; doi:10.1371/journal.pone.0304255)
Supplement: Attachment — Submitted filename: Response to reviewers- 5 April 2024.docx [file pone.0304255.s005.docx]

Response to reviewers: **PONE-D-23-33845R2**

Dear Muhammad Shahzad Aslam thank you for reviewing our systematic review protocol and your positive feedback.

We have addressed your comment by adding more information throughout the manuscript. Please see our response below and the revised manuscript uploaded.

|  | Comments | Response |
| --- | --- | --- |
| #1 | Explain authors prospect interpretation of expected findings, provide context, discuss implications, and suggest future directions. | Thank you for your critical comment. We have addressed your comment by adding more information throughout the manuscript. For example, on page #, context have been strengthened by rewording some sections:  On page #11”, we have included the following sentences. *This systematic review will provide an evidence base for determinants of effective HIV intervention strategies to inform policy makers and program planners to address HIV outcome inequalities among BWAD in HICs. This study will have significant importance in guiding context-based policy and program planning and implementation through summarizing finding on determinants of effective HIV interventions barriers and facilitators and key lessons learned to date from efforts to provide or access HIV interventions to address the HIV vulnerability and improve health to BWAD in HICs*.  On page 12, we have provided more information to strengthen implication of study results for our local, Canadian educational intervention module. |
| #2 | Highlight how their findings contribute to the current understanding of HIV prevention, treatment, and care among Black Women of African Descent in High-Income Countries (BWAD). | Thank you for your comment. Please see on page #5, now we have added more information*.*  *” Despite the multiple individual context-based studies focusing on the factors associated with HIV vulnerability or risk factors, there is limited evidence on the determinants of the effectiveness of HIV intervention to address the HIV inequality outcomes and impact among BWAD in HICs. There is no systematic review focusing on HIV interventions tailored to BWAD and determinants of effective HIV intervention to address the HIV inequality outcomes and impact among BWAD in HICs. This systematic review will inform practice and policy interventions for effectively addressing HIV and related healthcare as well as improving inequalities in HIV outcomes among BWAD and beyond in HICs. We will identify, evaluate, and summarize the findings of all relevant individual studies on HIV intervention among BWAD in HICs, thereby making the available evidence more accessible for policy and practice for tailored, innovative, and equitable approach to addressing HIV-related Health outcomes inequalities among BWAD and other racialized people.”* |
| #3 | Mention that the discussion section typically addresses the implications of the study's findings for theory, practice, and policy. Ask the authors to consider the practical implications of their findings for healthcare providers, policymakers, and researchers working in the field of HIV prevention and care for BWAD. | Thank you for your comments: Please see on page #16, now we have added more information about the implication of the study finding for policy and practice and researchers.  *“this systematic review will provide an evidence base on multilevel barriers, facilitators and lessons in HIV intervention to inform policy makers and program planners to practice equity driven HIV programs while addressing HIV outcome inequalities among BWAD and other racialized people in HICs. Also, this study will have significant importance in guiding context-based policy and implementation through summarizing finding on determinants of effective HIV interventions to address the HIV vulnerability and improve health to BWAD in HICs.”* |
| #4 | It's still important to address the limitations and strengths of the study that is expected. Planned it properly. | Thank you for bringing this to our attention. Please see on page #11, now we have added more information about the limitations and strengths of the study.  *“**Strengths and limitations of this study*  *Our study has multiple strengths. First, the best of our knowledge, no systematic review has examined the existing determinants of effectiveness of HIV prevention, treatment, and care interventions to address the HIV outcomes disparities and inequities among BWAD in HICs using quantitative and qualitative studies. Second, the research team has extensive experience in BWAD health and HIV studies and have multiple publications and work on BWAD health and HIV studies in HICs and beyond. Third, the use of a comprehensive search strategy and a range of databases in consultation with health science librarian to develop the protocol.*  *Limitation, it would be difficult to avoid publication bias because of the exclusion of secondary/grey literature; studies published in languages other than English; and exclusion of studies including population other than BWAD. “* |
