# Supplementary material for: Determinants of effective interventions for HIV prevention, treatment, and care to address inequitable HIV outcomes among Black Women of African Descent (BWAD) in High-Income Countries: Systematic review protocol
Source: PLoS One. 2024 Jun 11;19(6):e0304255. doi: 10.1371/journal.pone.0304255 (PMC11166283; doi:10.1371/journal.pone.0304255)
Supplement: Attachment — Submitted filename: Response to Reviewers-May 2, 2024.docx [file pone.0304255.s006.docx]

May 2, 2024- SR- Response to Reviewers'

PONE-D-23-33845R3

Determinants of effective interventions for HIV prevention, treatment, and care to address inequitable HIV outcomes among Black Women of African Descent (BWAD) in High-Income Countries: Systematic review protocol.

PLOS ONE

|  | Reviewers’ comments | Response |
| --- | --- | --- |
| 1 | 1.1. Firstly, it would be beneficial to provide more information about the process of participant selection to ensure the sample is representative of the population under study, thereby mitigating selection bias. | Thank you for this comment. We have clearly pre-defined selection criteria for the included articles based on PICO inclusion and exclusion criteria while adhering to the JBI SR guidelines. Please see this on page#7 |
|  | 1.2. Additionally, consider discussing strategies employed to minimize observer bias, such as blinding protocols or inter-rater reliability measures. Furthermore, please ensure that measurement instruments are validated and applied consistently across all study participants to mitigate the risk of measurement bias. | Thank you for this comment. For this systematic review, covidence has a full blinding for screening and screening conflict resolution. In addition to the strategies for uploading articles into Covidence and article screening outlined on page #9, we have added the information to strengthen this section.  We have this section to include steps taken to minimize observer bias and improve inter-rater reliability. E.g. Each title, abstract and full article will be screened by two team members and disagreements will be resolved by a third reviewer. |
|  | 1.3. Finally, transparency in reporting is crucial. Ensure that all relevant findings, regardless of significance, are included in the manuscript to avoid reporting bias. Addressing these biases will strengthen the validity and reliability of your study findings. | Thank you for this comment.  We will report the findings in accordance with the reporting guidance provided in the PRISMAs statement. Covidence offers tools and a workflow for all data extraction that would be necessary for the final SR reporting. It produces a PRISMA flow diagram and is able to export additional information.  Please see on page# 7. |
| 2 |  |  |
|  | 2.1. As I review your protocol, I believe that emphasizing the practical implications of your findings within the discussion section would greatly enhance the relevance and impact of your study. Given the significance of your research topic, it is imperative to clearly articulate how the identified determinants of effective interventions can be translated into actionable strategies to improve HIV outcomes for BWAD. | Thank you for this comment.  We have described how the finding will be policies and interventions. Our study will guide the multilevel policy and intervention using multiprong approach including targeted behavioral, biomedical, and structural interventions guided by guiding equity-based health policy and intervention to address the HIV vulnerability and improve health to BWAD in HICs.  Please see on page #11 |
|  | 2.2. I encourage you to dedicate a section of the discussion to explicitly outline the practical implications of your findings. | Can be combined with 2.1 |
|  | 2.4. Consider addressing questions such as: How can the identified determinants inform the development and implementation of targeted interventions for BWAD? | Can be combined with 2.1 |
|  | 2.5. What policy changes or healthcare practices could be influenced by your findings to address disparities in HIV outcomes? | Thank you for your comments. Our finding will inform BWAD and other racialized community based HIV and health policy and practice using multilevel determinants and using multiprong approach including targeted behavioral, biomedical, and structural interventions to address the BWAD health inequity challenges. Please see on page 11. |
|  | 2.6. Providing concrete examples or recommendations for stakeholders and policymakers will strengthen the applicability of your research in real-world settings.  By integrating a discussion of practical implications, your study will not only contribute to the academic literature but also provide valuable guidance for addressing healthcare disparities and improving HIV-related outcomes among BWAD in high-income countries. | Thank you for bringing this to our attention. Our study finding will guide the multi level and sectoral stakeholder including public health, community-based organizations and approaches, and nongovernmental civil society organization BWAD HIV and health policy and practice in HICs using multiprong approach including targeted behavioral, biomedical, and structural interventions to address the BWAD health inequity challenges. Please see on page #11 |
| 3 | I am not satisfied with your current method on Assessment of risk of bias in included studies.  Please add Cochrane Collaboration's Tool for Assessing Risk of Bias, Newcastle-Ottawa Scale (NOS) and Jadad Scale. | Thank you for your comment.  Given the study methods we will use JBI’s critical appraisal tools to assess the included studies. We have added the following information on page # to clarify our assessment method.  JBI’s critical appraisal tools helps to assess varieties of study design including cross sectional studies, cohort studies, economic evaluations, prevalence studies, qualitative research. The JBI critical appraisal tool includes multiple questions addressing the internal validity and risk of bias of study designs, particularly confounding, selection, and information bias, in addition to the importance of clear reporting. |
|  |  |  |
| 4 | 4- Explain Joanna Briggs Institute  critical appraisal checklists(40). | Thank you for your comment. Now, we have added more description.  Please see on page# 10.  JBI’s critical appraisal tools assist in assessing the methodological quality of a study and to determine the extent to which a study has addressed the possibility of bias in its design, conduct and analysis including the trustworthiness, relevance, and results of studies. |
| 5 | 5.1. Detail how Covidence will be utilized to assess the risk of bias in the included studies. Describe the criteria and tools that will be used for evaluating study quality and risk of bias, and explain how Covidence will facilitate this process. | Thank you for your comment.  We will not use covalence’s default quality assessment template. JBI’s critical appraisal tools helps to assess the included studies, please see on page#10.  For certainty of evidence, we will use the GRADE-CERQual for qualitative studies and GRADE approach for the quantitative studies. Please see on page #9. |
|  | 5.2. Describe how data extracted from the included studies will be synthesized and analyzed using Covidence. Outline any planned approaches for meta-analysis, subgroup analysis, or qualitative synthesis, and specify how Covidence will support these analyses. Provide the flow diagram. | Thank you for your comments. We will use the Covidence for the screening phase only. We will adapt a data collection form based on the needs of the review from a standardized data extraction form by the Cochrane library.  Please see on page #9 |
| 6 | Develop the PICO TABLE. | Please see on page#; We have included this as a narrative on page. |
|  | It is important to write discussion with previous literature. Please explain why this review is needed | Thank you for your comments. We have added more information on page # 11.  First, we will summarize the key findings from the research and link them to the initial research question. Second, we will place the findings in context. Through going back to the literature and analyzing how the results fit in with previous research. |

1-I have carefully reviewed your manuscript and find it to be a valuable contribution to the field. However, I would like to bring to your attention some potential biases that may need to be addressed.

1.4. Firstly, it would be beneficial to provide more information about the process of participant selection to ensure the sample is representative of the population under study, thereby mitigating selection bias.
1.5. Additionally, consider discussing strategies employed to minimize observer bias, such as blinding protocols or inter-rater reliability measures. Furthermore, please ensure that measurement instruments are validated and applied consistently across all study participants to mitigate the risk of measurement bias.
1.6. Finally, transparency in reporting is crucial. Ensure that all relevant findings, regardless of significance, are included in the manuscript to avoid reporting bias. Addressing these biases will strengthen the validity and reliability of your study findings. Thank you for considering these suggestions, and I look forward to seeing how you incorporate them into your manuscript.

2- I commend you on the comprehensive systematic review protocol titled "Determinants of Effective Interventions for HIV Prevention, Treatment, and Care to Address Inequitable HIV Outcomes Among Black Women of African Descent (BWAD) in High-Income Countries." Your protocol lays a strong foundation for investigating crucial aspects of HIV prevention, treatment, and care among BWAD in high-income countries.

2.1. As I review your protocol, I believe that emphasizing the practical implications of your findings within the discussion section would greatly enhance the relevance and impact of your study.

2.2. Given the significance of your research topic, it is imperative to clearly articulate how the identified determinants of effective interventions can be translated into actionable strategies to improve HIV outcomes for BWAD.

2.3. I encourage you to dedicate a section of the discussion to explicitly outline the practical implications of your findings.

2.4. Consider addressing questions such as: How can the identified determinants inform the development and implementation of targeted interventions for BWAD?

2.5. What policy changes or healthcare practices could be influenced by your findings to address disparities in HIV outcomes?

2.6. Providing concrete examples or recommendations for stakeholders and policymakers will strengthen the applicability of your research in real-world settings.

2.7. By integrating a discussion of practical implications, your study will not only contribute to the academic literature but also provide valuable guidance for addressing healthcare disparities and improving HIV-related outcomes among BWAD in high-income countries.

3-I am not satisfied with your current method on Assessment of risk of bias in included studies. Please add Cochrane Collaboration's Tool for Assessing Risk of Bias, Newcastle-Ottawa Scale (NOS) and Jadad Scale.

4- Explain Joanna Briggs Institute

critical appraisal checklists(40).

5.Detail how Covidence will be utilized to assess the risk of bias in the included studies. Describe the criteria and tools that will be used for evaluating study quality and risk of bias, and explain how Covidence will facilitate this process. Describe how data extracted from the included studies will be synthesized and analyzed using Covidence. Outline any planned approaches for meta-analysis, subgroup analysis, or qualitative synthesis, and specify how Covidence will support these analyses. Provide the flow diagram.

6- Develop the PICO TABLE.

7-It is important to write discussion with previous literature. Please explain why this review is needed
